# Tetrahedral framework nucleic acid loaded with glabridin: A transdermal delivery system applicated to anti‐hyperpigmentation

**DOI:** 10.1111/cpr.13495

**Published:** 2023-05-03

**Authors:** Jiajun He, Wen Chen, Xingyu Chen, Yu Xie, Yuxuan Zhao, Taoran Tian, Bin Guo, Xiaoxiao Cai

**Affiliations:** ^1^ State Key Laboratory of Oral Diseases, National Clinical Research Center for Oral Diseases, West China Hospital of Stomatology Sichuan University Chengdu China; ^2^ Department of Stomatology First Medical Center of Chinese PLA General Hospital Beijing China

## Abstract

Topical application of tyrosinase inhibitors, such as hydroquinone and arbutin, is the most common clinical treatment for hyperpigmentation. Glabridin (Gla) is a natural isoflavone that inhibits tyrosinase activity, free radical scavenging, and antioxidation. However, its water solubility is poor, and it cannot pass through the human skin barrier alone. Tetrahedral framework nucleic acid (tFNA), a new type of DNA biomaterial, can penetrate cells and tissues and can be used as carriers to deliver small‐molecule drugs, polypeptides, and oligonucleotides. This study aimed to develop a compound drug system using tFNA as the carrier to transport Gla and deliver it through the skin to treat pigmentation. Furthermore, we aimed to explore whether tFNA–Gla can effectively alleviate the hyperpigmentation caused by increased melanin production and determine whether tFNA–Gla exerts substantial synergistic effects during treatment. Our results showed that the developed system successfully treated pigmentation by inhibiting regulatory proteins related to melanin production. Furthermore, our findings showed that the system was effective in treating epidermal and superficial dermal diseases. The tFNA‐based transdermal drug delivery system can thus develop into novel, effective options for non‐invasive drug delivery through the skin barrier.

## Graphical abstract

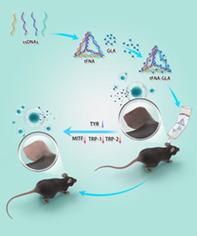

## 1 INTRODUCTION

Hyperpigmentation, a common skin disorder in humans, has no standard treatment.^1^, ^2^, ^3^ The most common types of hyperpigmentation include melasma, freckles, senile plaques, and postinflammatory hyperpigmentation.^4^, ^5^, ^6^, ^7^, ^8^ Although the clinical and pathological manifestations of each type differ, increased melanin production and uneven distribution are the leading causes of hyperpigmentation and are related to hormones, inflammation, and ultraviolet (UV) exposure.^9^, ^10^, ^11^, ^12^, ^13^

Melanin, the primary pigment in human hair and skin, is synthesized in the melanosomes of melanocytes and protects the skin from the harmful effects of UV exposure and other environmental factors.^14^, ^15^, ^16^ Tyrosinase (TYR) is critical in melanin synthesis. TYR and TYR‐related proteins (TRP‐1 and TRP‐2) are transported from the nucleus to melanosomes at an early stage and catalyse the synthesis of melanin.^17^, ^18^, ^19^ Specifically, melanin synthesis in the human body begins with l‐tyrosine, which is finally converted to melanin through complex processes. In this pathway, TYR converts l‐tyrosine to dopaquinone (DQ). The subsequent melanin synthesis process can proceed spontaneously under physiological conditions. Finally, two types of melanin (eumelanin and pheomelanin) are produced after DQ undergoes a series of biochemical reactions.^20^ Thus, TYR plays a crucial role in melanin synthesis.

Topical TYR inhibitors are commonly used in the treatment of hyperpigmentation.^21^, ^22^ Currently available treatments for hyperpigmentation include hydroquinone (HQ) and arbutin, and other treatments.^23^, ^24^ Glabridin (Gla) is a natural isoflavone with potential application in skin lightening, photoaging treatment, and alleviation of pigmentation and erythema, due to considerable inhibition of TYR activity, free radical scavenging, and antioxidant activity.^25^, ^26^, ^27^, ^28^ However, Gla has poor water solubility and low bioavailability, and penetration of the skin barrier without the synergistic effect of a carrier is challenging.

As a novel bionanomaterial, tetrahedral framework nucleic acid (tFNA) has attracted extensive attention in drug delivery, disease treatment, and biomedical imaging research due to its structural stability, sufficient flexibility, and biocompatibility.^29^, ^30^, ^31^, ^32^, ^33^ The stable tetrahedral structure of tFNA facilitates penetration of the cell membrane barrier through unique interactions with cells and has the potential to carry small‐molecule drugs.^34^, ^35^, ^36^, ^37^ In previous studies, tFNA delivered small‐molecule drugs, polypeptides, and oligonucleotide drugs.^38^, ^39^, ^40^ Relying on its biological and structural properties for cell penetration, tFNA can adopt lower electrostatic repulsion to enter cells through cavernous protein internalization.^36^, ^41^, ^42^, ^43^ Further studies have also found that tFNA can pass through the skin barrier smoothly through skin attachments and has a high tissue penetration capacity.^31^ Thus tFNA can reach deep tissues in a non‐invasive manner.

Here, we aimed to develop a compound drug system using tFNA as the carrier to transport Gla and deliver it through the skin to treat pigmentation. Furthermore, we explored whether tFNA–Gla can effectively alleviate the hyperpigmentation caused by increased melanin production by inhibiting TYR activity. Since tFNA has antioxidant and anti‐inflammatory effects, we also sought to determine whether tFNA–Gla exerts substantial synergistic effects during treatment. This study would lay a foundation for further exploring the application potential of tFNA in percutaneous therapy.

## 2 MATERIALS AND METHODS

### 2.1 Fabrication and authentication of tFNA–Gla

Self‐assembled with specifically designed sequences (Sangon Co. Ltd., Shanghai, China) in TM buffer (50 mM MgCl_2_ and 10 mM Tris–HCl, pH 8.0), tFNA was synthesized from four single DNA strands (ssDNAs) after a series of heating procedures (95°C for 10 min and 4°C for 20 min)^44^; the four ssDNAs were in equimolar amounts (1 μM). The ssDNA sequences are presented in Table 1. Then tFNA (250 nm) was mixed with various concentrations of Gla (40, 80,120, 160 and 200 μM) and stirred for 6 h. The successful synthesis of tFNA–Gla was confirmed using fluorescence.

**TABLE 1:** Sequence of Each ssDNA.

ssDNA	Direction	Sequence
S1	5′–3′	ATTTATCACCCGCCATAGTAGACGTATCACCAGGCAGTTGAGACGAACATTCCTAAGTCTGAA
S2	5′–3′	ACATGCGAGGGTCCAATACCGACGATTACAGCTTGCTACACGATTCAGACTTAGGAATGTTCG
S3	5′–3′	ACTACTATGGCGGGTGATAAAACGTGTAGCAAGCTGTAATCGACGGGAAGAGCATGCCCATCC
S4	5′–3′	ACGGTATTGGACCCTCGCATGACTCAACTGCCTGGTGATACGAGGATGGGCATGCTCTTCCCG
Cy5‐S1	5′–3′	Cy5ACGGTATTGGACCCTCGCATGACTCAACTGCCTGGTGATACGAGGATGGGCATGCTCTTCCCG

### 2.2 Characterization of tFNA–Gla

To observe and analyse the tetrahedral spatial structure and nanosize of tFNA and tFNA–Gla, atomic force microscopy (AFM; Shimadzu, Japan) and transmission electron microscopy (TEM; Tecnai G2 F20 S‐TWIN, Japan) were used. To detect the zeta potentials and particle sizes of the tFNA and tFNA–Gla, dynamic light scattering (DLS; ZETASIZER NANO ZS90, England) was used.

### 2.3 Cell culture and groups

Murine melanoma B16 cells were cultured in an incubator (5% CO_2_, 37°C) according to the recommendations of ScienCell. The culture medium consisted of high‐glucose DMEM, 10% foetal bovine serum (HyClone, USA), and 1% penicillin–streptomycin solution (HyClone). Subsequently, B16 cells were randomly divided into five groups: blank, control (Ctrl), Gla, tFNA, and tFNA–Gla complex (TGC). The cells were then inoculated in a culture flask. The culture media were changed every 48 h.

### 2.4 Uptake of tFNA–Gla by melanoma cells

To examine the success of the tFNA–Gla system, we attached Cy‐5 fluorescent molecules to a single strand (Cy5‐S1) of the nucleic acid. B16 cells were then treated with the Cy5‐labelled complex for 2–8 h and collected for examination. The ratio of the number of Cy5‐labelled cells to the total number of cells was detected using a flow cytometer (CytoFLEX, Beckman Coulter Inc., Brea, USA). Confocal microscopy (Olympus, Tokyo, Japan) was performed to capture images of the Cy5‐labelled tFNA–Gla distributed in the cells.

### 2.5 Cell viability assays

The B16 cells were pre‐processed with various cytotoxic ratios of tFNA–Gla (tFNA: Gla = 1:20–1:800; the concentration of tFNA was 250 nM) for 12 and 24 h. A cell counting kit 8 (MedChemExpress, NJ, USA) was used to test the cytotoxicity of tFNA–Gla. The optical density (OD) values of the samples were recorded at 450 nm to determine cell viability.

### 2.6 Measurement of melanin content

To measure melanin content, first, the B16 cells were pre‐processed with tFNA–Gla. The precipitate was dissolved in 2 mL of EtOH–ether solution (3:1) and centrifuged. Next, the precipitate was resuspended in 2 mL of ether, followed by centrifugation for 10 min. The precipitate was then air‐dried and resuspended in NaOH (0.5 mL) at 80°C for 1 h. The absorbance of the samples was measured at 415 nm.

### 2.7 Western blotting analysis

The levels of TYR, microphthalmia‐related transcription factor (MITF), TRP‐1, and TRP‐2 were detected using western blotting. First, the B16 cells were seeded into six‐well plates (10^6^ cells/well). After treatment with Gla, tFNA or tFNA–Gla, protein samples were harvested from the different groups using a cell protein extraction reagent (KeyGen Biotech Co. Ltd., Nanjing, China). After separating the proteins using 5% SDS‐PAGE, the resulting protein bands were transferred onto poly(vinylidene fluoride) membranes via electroblotting. Primary antibodies (anti‐TYR, anti‐TRP‐1, anti‐TRP‐2 and anti‐MITF) were purchased from Abcam (Cambridge, UK). The membranes were incubated with the primary antibodies overnight. The next day, the membranes were incubated with the appropriate secondary antibodies (Beyotime, Shanghai, China). Finally, an ECL reagent (Millipore, MA, USA) was used to visualize the protein bands.

### 2.8 Immunofluorescence staining

Immunofluorescence staining was used for sample detection. The samples were blocked with 5% sheep serum for 1 h and incubated with the primary target antibodies anti‐TYR and anti‐MITF at 4°C overnight. The next day, the samples were rinsed thrice with PBS and incubated with a secondary antibody (1:500; Invitrogen, Carlsbad, CA, USA) for 1 h. The nuclei and cytoskeletons were stained using DAPI and phalloidin, respectively. Finally, the samples were stored in 10% glycerol and observed using a laser confocal microscope (Olympus, Tokyo, Japan).

### 2.9 Animal experiments

The Medical Ethics Committee of West China Hospital of Stomatology, Sichuan University reviewed and approved the animal experiments (approval number: WCHSIRB‐D‐2023‐021). We purchased C57BL/6J mice (female, 20 g, 4‐week‐old) from GemPharmatech (Nanjing, China) and bred them under standard environmental conditions. The mice were randomly assigned into six groups: Blank (NS, no UVB), Ctrl (NS, UVB), 4% HQ (4%HQ, UVB), Gla (40 μM, UVB), tFNA (250 nM, UVB), and tFNA‐Gla (tFNA:250 nM, Gla:40 μM). To explore the capacity of tFNA–Gla to reduce pigment accumulation in mice, we constructed a mouse model of melasma using UVB irradiation (100 mJ/cm^2^/day, every other day for 4 weeks).^45^ After the establishment of the model (4 weeks), the six components were mixed separately with Aquaphor (Eucerin, Germany) at a ratio of 1:1. Tegaderm (3M, USA) was then applied to the ear to confirm that the drug remained in the treatment area until the next dressing change. The mice received the corresponding topical drug on the ear skin daily for 14 consecutive days (once a day). During the modelling and drug administration period, photographs were obtained to record the colour and related texture of mouse ears in each group. Adobe Photoshop 2020 was used for colour analysis. Photographs of the ears of each mouse were checked automatically, and the average RGB values and brightness curves were recorded. After the mice were sacrificed by spinal dislocation, their ears were collected and stained with haematoxylin and eosin (H&E) and Masson's trichrome technique.

### 2.10 Statistical analysis

The GraphPad Prism software version 8.0.2 (San Diego, USA) was used for statistical analysis. The statistical methods employed for comparisons between the groups were Student's *t*‐test and one‐way analysis of variance. Quantitative data are presented as means ± standard deviations (*n* ≥ 3). All differences with *p* values <0.05 were considered significant.

## 3 RESULTS AND DISCUSSION

### 3.1 Synthesis and characterization of tFNA–Gla

**FIGURE 1:**
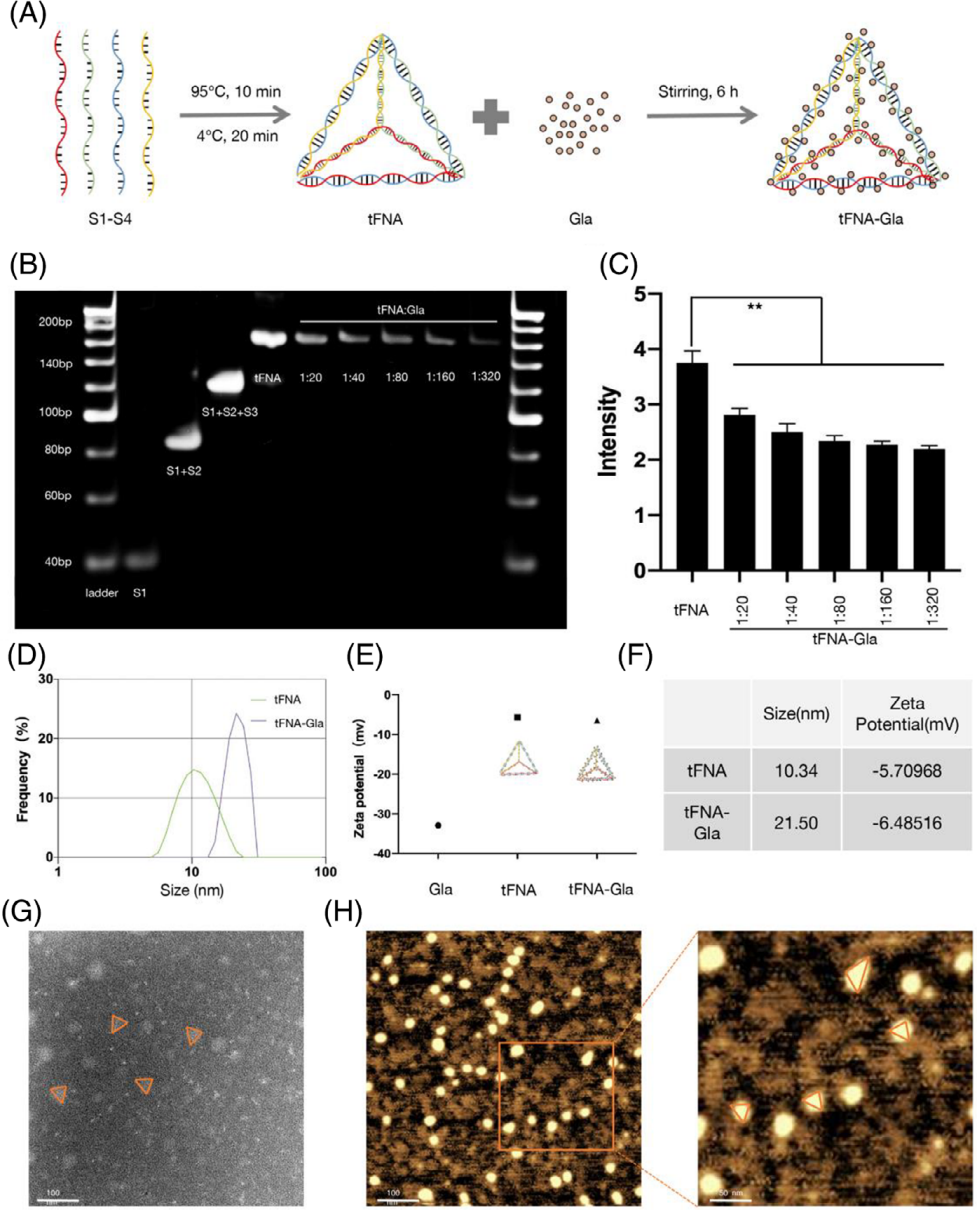
Synthesis and characterization of tFNA‐Gla. (A) Schematic of the synthesis process of tFNA and tFNA‐Gla. (B) Confirmation of the successful synthesis of tFNA and tFNA‐Gla by PAGE. Lane 1, S1; Lane 2, S1 + S2; Lane 3, S1 + S2 + S3. Lane 4, tFNA; Lane 5, tFNA: Gla = 1:20; Lane 6, tFNA: Gla = 1:40; Lane 7, tFNA: Gla = 1:80; Lane 8, tFNA: Gla = 1:160; Lane 9, tFNA: Gla = 1:320. (C) The intensity of Gel‐Red fluorescence of tFNA‐Gla to Gel‐Red fluorescence of tFNA with different ratios of Gla (tFNA: Gla = 1:20–320). (D) Molecular size of tFNA and tFNA‐Gla measured by DLS. (E) Zeta potential of Gla, tFNA and tFNA‐Gla measured by DLS. (F) The comparison of the size result of tFNA and tFNA‐Gla. (G) The TEM image of tFNA‐Gla (scale bar: 100 nm). (H) The AFM image of tFNA‐Gla (Scale bar: 100 nm). (statistical analysis: *p < 0.05; **p < 0.01; ***p < 0.001).

Figure 1A shows the process of tFNA synthesis using the four ssDNAs listed in Table 1. Different ratios of Gla and tFNA (tFNA: Gla = 1:20–320) were stirred for 6 h to form complexes. SDS‐PAGE was used to verify TGC synthesis and to characterize the complexes (Figure 1B). The tFNA and tFNA–Gla were stained using GelRed. In the PAGE, the width of the different complex bands decreased with increasing concentrations of Gla. Besides, in the GelRed fluorescence competition experiment, the fluorescence intensity of GelRed decreased with increasing Gla concentration. This result illustrates that Gla and GelRed compete for binding sites on tFNA (Figure 1C).

The size and zeta potential of the particles was determined using DLS was used (Figure 1D,E). The mean size of tFNA–Gla was 21.50 nm (Figure 1D,F), and the mean zeta potential value was −6.49 mV (Figure 1E,F).

AFM and TEM images showed that tFNA–Gla had a size of 15 nm, with a tetrahedral structure (Figure 1G,H).

Based on these results, we demonstrated for the first time the successful construction of a tetrahedral model of TGC using intercalation binding of Gla to nucleic acid duplexes. In subsequent experiments, we studied whether this model can be used as a novel transdermal drug delivery system.

### 3.2 Delivery function of tFNA–Gla

Studies have reported that tFNA has unique structural properties and can enter cells.^46^ To explore the ability and efficiency of nucleic acids with a tetrahedral backbone to enter B16 cells, confocal microscopy and flow cytometry were used to detect the uptake of Cy5‐labelled tFNA and tFNA–Gla.

**FIGURE 2:**
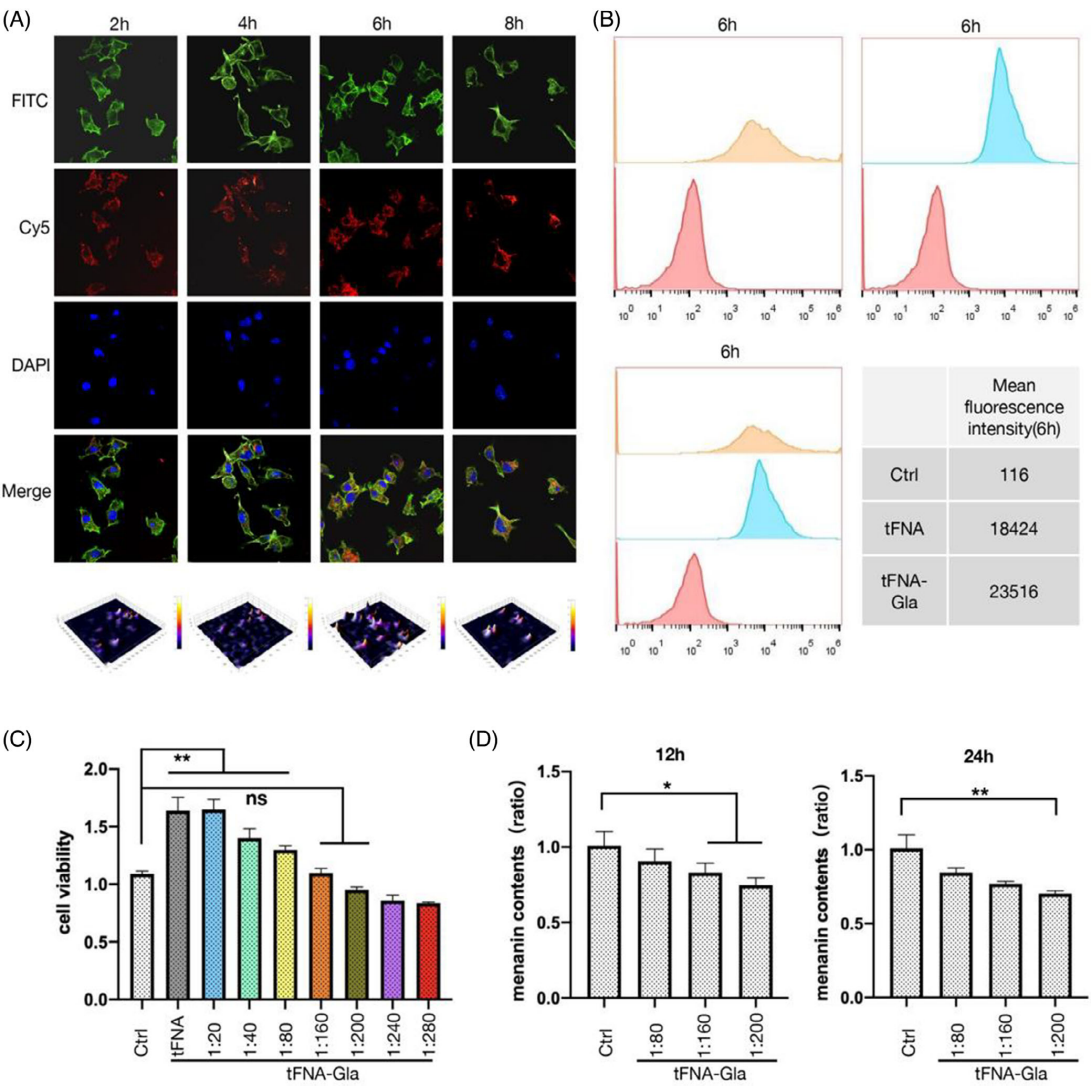
tFNA‐Gla enters b16 cells to inhibit the synthesis of melanin. (A) Cellular uptake of the Cy5‐tFNA, as determined by immunofluorescence staining (blue: nucleus; red: Cy5‐labelled tFNA; green: cytoskeleton). (B) Quantitative detection and analysis of the intracellular tFNA and tFNA‐Gla levels using flow cytometry. (C) The cell viability of the b16 cell was evaluated after exposure to tFNA‐Gla of different concentrations. (D) The effect of different concentrations of tFNA‐Gla on melanin synthesis of the B16 cell after 12 and 24 h. (statistical analysis: *p < 0.05; **p < 0.01; ***p < 0.001).

Confocal microscopy images show that fluorescence intensity decreased after 6 h (Figure 2A), whereas the highest tFNA–Gla fluorescence intensity was observed at 6 h (Figure 2B). However, after 8 h, Cy5‐labelled tFNA and tFNA–Gla were widely distributed in the cytoplasm. These results indicate that tFNA–Gla has good cell‐entry performance.

In addition, cell viability assays revealed that a tFNA: Gla ratio of 1:160 achieved maximum drug loading without inhibiting cell proliferation. At the same time, tFNA at a concentration of 250 nM effectively promoted cell proliferation (Figure 2C). Thus, 40 μM tFNA–Gla was determined to possess good biocompatibility properties.

### 3.3 Inhibitory activity of tFNA–Gla on melanin synthesis

Melanocytes produce melanin in the basal layer.^47^ Melanosomes are the organelles that synthesize melanin in melanocytes.^48^ During maturation, melanosomes gradually move to the epidermis as keratinocytes mature, contributing to the colour of the skin.^49^, ^50^, ^51^, ^52^ Therefore, if the tFNA–Gla complex can reduce melanin production, it could be a potential strategy for treating hyperpigmentation.

After treatment with drugs for 12 and 24 h, B16 cells were collected, and the intercellular melanin was dissolved with NaOH. The melanin content is represented by the absorbance of the solution at 415 nm. Melanin content was significantly reduced in the groups treated with Gla and TGCs compared to that in the untreated and tFNA groups (Figure 2D). These results further confirm that Gla has an inhibitory effect on cellular melanin synthesis.

### 3.4 tFNA–Gla reduces the activity of proteins related to melanin synthesis

TYR, MITF, TRP‐1 and TRP‐2 play important roles in melanin synthesis. After activation, TYR converts l‐tyrosine into dopaquinone, which can be used in subsequent reactions to produce melanin.^53^, ^54^, ^55^ During this process, factors secreted by keratinocytes bind to melanocyte receptors and initiate melanin synthesis, including cAMP stimulation.^56^, ^57^ cAMP increases the expression of MITF; then upregulates the expression of TYR, TRP‐1 and TRP‐2; finally promotes melanin synthesis in melanocytes.^58^

**FIGURE 3:**
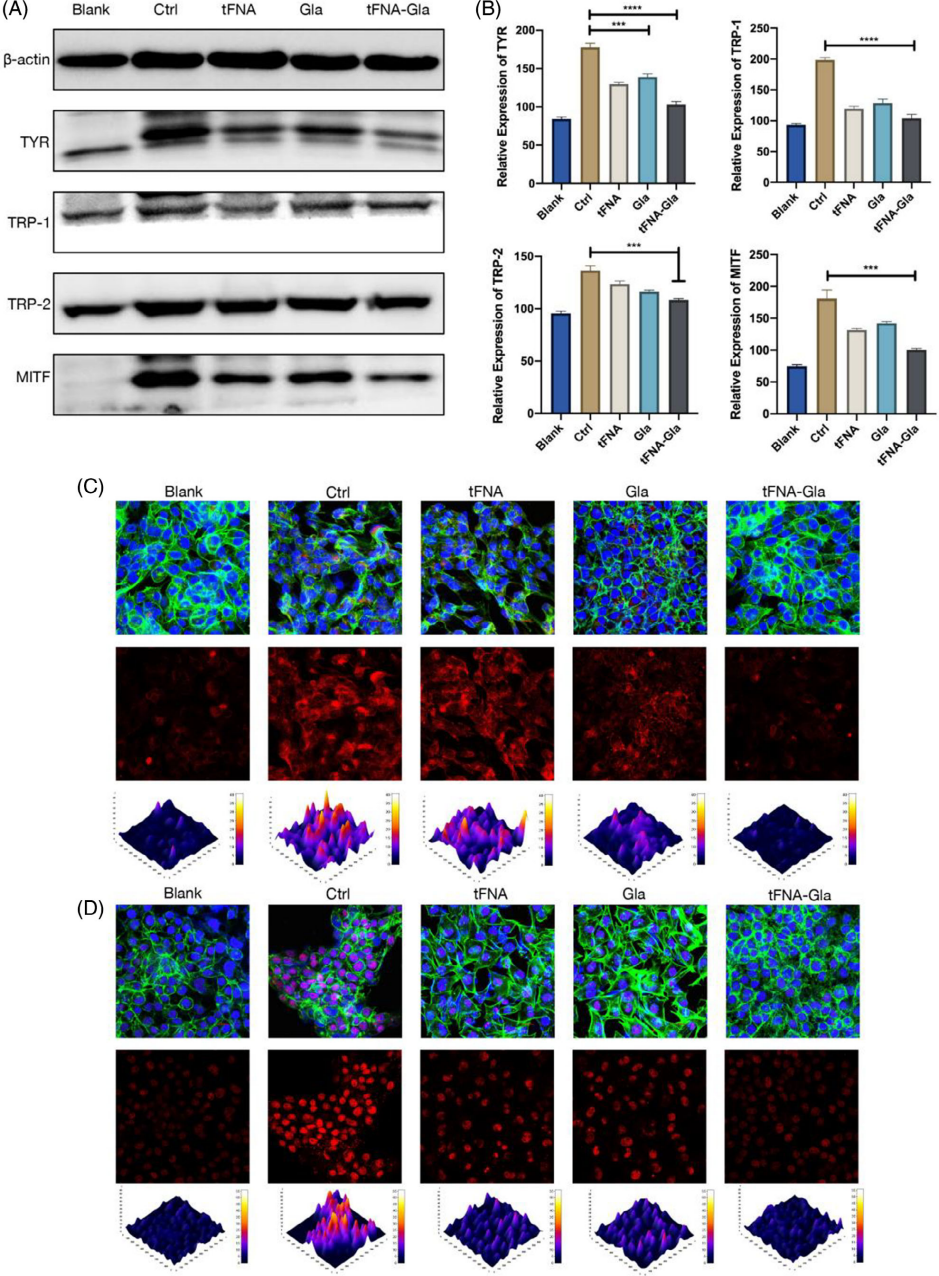
Modulation of key signalling molecules in melanin synthesis. (A) Western blotting results of TYR, TRP‐1, TRP‐2 and MITF expression level. (B) The expression levels of the above proteins were quantitatively analysed. (C) Immunofluorescence images of TYR expression in cells after different treatments (cytoskeleton: green; nucleus: blue; TYR: red; 3D thermal imaging: reconstruction of fluorescence intensity of TYR). Scale bar: 100 μm. (D) Immunofluorescence images of MITF expression in cells after different treatments (cytoskeleton: green; nucleus: blue; MITF: red; 3D thermal imaging: reconstruction of fluorescence intensity of MITF). Scale bar: 100 μm (statistical analysis: *p < 0.05; **p < 0.01; ***p < 0.001).

Western blotting revealed that the expression of all four proteins in the TGC group was significantly lower than that in the Ctrl group (Figure 3A,B), indicating that the TGC group produced fewer melanin synthesis‐related proteins over the same period. Similarly, the expression of all four proteins in the TGC group was lower than that in the Gla group. Previous studies have found that tFNA has a good cell penetration capacity and can enter cells by lowering electrostatic repulsion. We speculate that the present results are due to these properties.

We also measured the expression levels of TYR and MITF using immunofluorescence after treating B16 cells with tFNA–Gla. A stronger anti‐TYR fluorescence signal was observed in the tFNA–Gla than in the Ctrl group (Figure 3C). A similar result was observed for MITF (Figure 3D). These results indicate that tFNA–Gla inhibited the production and activity of TYR and thereby limited melanin production.

### 3.5 In vivo pigmentation model

**FIGURE 4:**
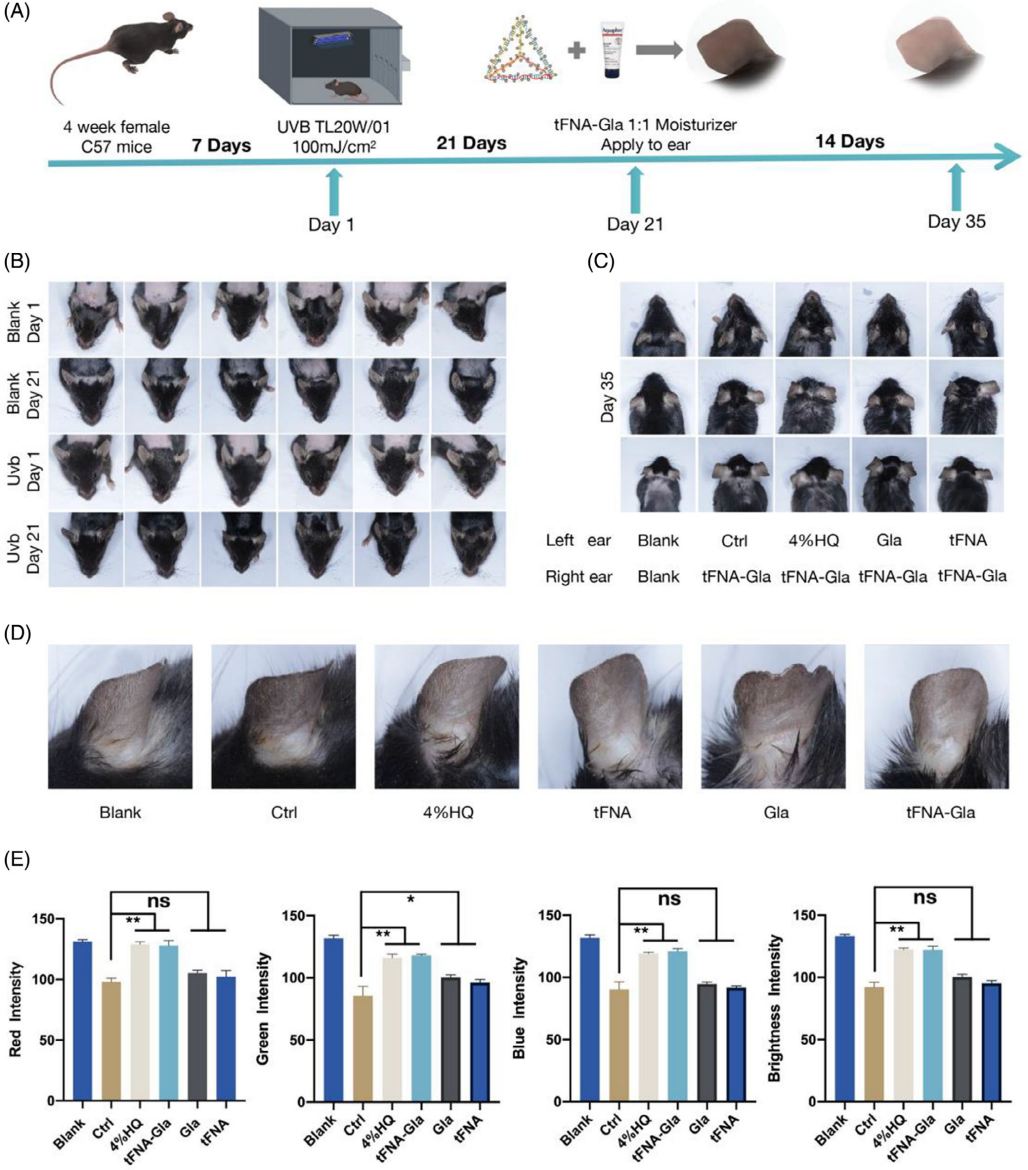
The tFNA‐Gla treatment of hyperpigmentation through transdermal drug delivery system. (A) Schematic diagram outlining pre‐treatment with the tFNA‐Gla and the establishment of an in vivo animal system. (B) Establishment of animal model of hyperpigmentation. (C) Photographs illustrate the skin lightening effect of tFNA‐Gla after topical application for 2 weeks on UVB‐irradiation‐induced hyperpigmentation in C57BL/6 mice. (D) Representative ear photographs of mice in different groups in (C) after treatment. (E) Adobe Photoshop 2020 was used to automatically identify the ear skin RGB parameters and the brightness curve information to make the statistical analysis (statistical analysis: *p < 0.05; **p < 0.01; ***p < 0.001).

To study one of the most common pigmentation conditions, melasma models have been widely used in scientific research.^59^, ^60^ Since mouse ear skin contains more melanocytes than trunk skin, the ear skin of C57BL/6J mice has been commonly used in melanin‐related studies.^61^ To verify the inhibitory effect of tFNA–Gla on pigmentation, we collected ear skin samples from all the mice (Figure 4A) in the melasma model (Figure 4B) after 14 days of induction. As shown in Figure 4C, after melasma induction, the mice were assigned to six groups (see Section 2.4), including self‐control (left and right ears).

The tFNA–Gla group exhibited a lower average RGB value than the Ctrl group (Figure 4E); this result is consistent in the images of representative ears (Figure 4C,D). A similar therapeutic efficacy was achieved using the commercially available TYR inhibitor 4% HQ (Figure 4E). Since Gla cannot be delivered transdermally, the Gla group did not exhibit a significant reduction in colour value compared with the Ctrl group (Figure 4E). Meanwhile, tFNA did not perform well in these in vivo experiments, reflecting the need for a compound drug system.

**FIGURE 5:**
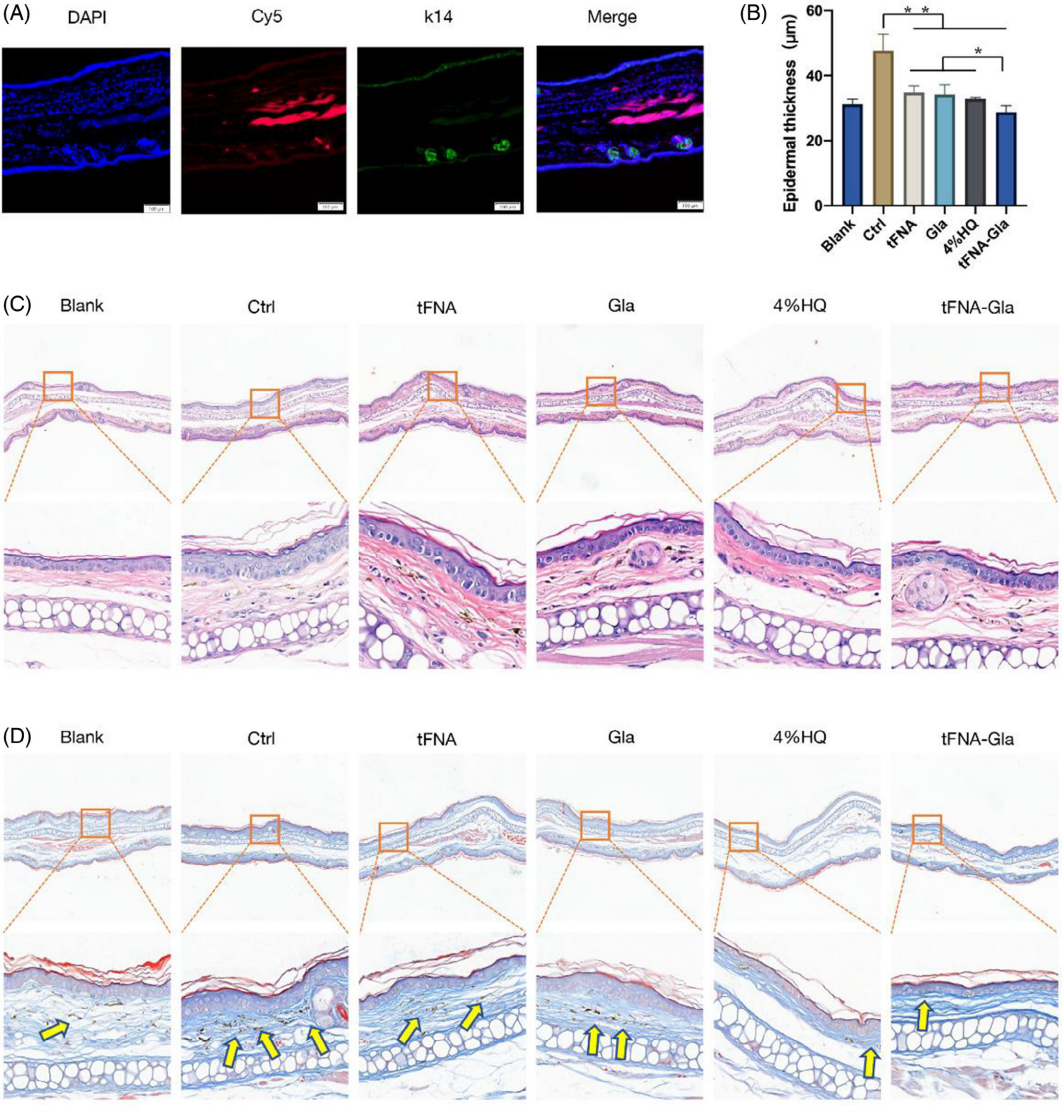
Evaluation of the anti‐hyperpigmentation treatment effect at the histological level. (A) The tissue penetration ability of cy5‐labelled tFNA‐Gla in C57BL/6 mice (blue: DAPI, green: K14, red: Cy5). (B) Statistical analysis of epidermal thickness. (C) Effect of tFNA‐Gla on UVB‐irradiation‐induced histopathological changes in C57BL/6 mouse ear skin. Tissue morphology was visualized through haematoxylin and eosin (H&E) staining. UVB irradiation caused hyperplasia in the skin, whereas topical application of tFNA‐Gla once a day to the ear skin reduced the thickness of the epidermis. (D) Effect of tFNA‐Gla on UVB‐irradiation‐induced hyperpigmentation in C57 mice ear. Melanin pigments (as indicated by the arrows) were stained blank by Fontana‐Masson staining. Topical application of tFNA‐Gla reduced melanin pigments (statistical analysis: *p < 0.05; **p < 0.01; ***p < 0.001).

We also investigated drug penetration in the mouse model. After topical application of Cy5‐labelled tFNA–Gla for 24 h, a strong fluorescence signal in the epidermis was evident in the histological images (Figure 5A). H&E staining is used to observe cell morphology and possible signs of inflammation, which often leads to the thickening of the epidermis (Figure 5C). Our results showed reduced skin increasing in the tFNA and TGC groups compared to that in the HQ group (Figure 5B). This result demonstrates that, regarding skin anti‐inflammation, a drug system with tFNA as the carrier may be an effective option. In contrast, products with high concentration of HQ currently available on the market may have more dramatic side effects. Figure 5D shows skin sections stained with Masson's trichrome, in which the melanin content in the tFNA–Gla group was significantly reduced; this effect was comparable to that of the 4% HQ group.

## 4 CONCLUSIONS

In this study, a tFNA‐based transdermal drug delivery system (TGC) was successfully synthesized. We confirmed the therapeutic effect of tFNA–Gla on pigmentation and examined the possible underlying mechanisms. The entire process was realized due to the excellent transcutaneous penetration capacity of the biological carrier tFNA. The intercalation of Gla in the double strand of nucleic acids formed a stable composite, TGC, which addressed the problem of inadequate Gla penetration of the skin barrier when Gla was used alone.

TGC deregulated melanin synthesis by inhibiting the expression of TYR and regulating the TRP‐1/TRP‐2/MITF signalling pathway. Their synergistic effect reduced melanin production in the epidermal cells and ultimately alleviated excessive pigment accumulation in the skin. However, whether TGC plays a role in melanin synthesis through other mechanisms requires further exploration.

Previously, we confirmed that tFNA can be used as a carrier to deliver small‐molecule drugs, polypeptides, and oligonucleotide drugs. Our research findings confirm that tFNA‐based transdermal drug delivery systems can become a new strategy for non‐invasive drug delivery through the skin. The TGC transdermal drug delivery system successfully delivered the small molecule Gla to the superficial dermis of mice, achieving an excellent therapeutic effect. The satisfactory tissue‐penetration capacity of tFNA indicates that it has extensive application as a carrier for treating epidermal and superficial dermal diseases. In the future, we will focus on tapping the massive potential of tFNA in the transdermal delivery mode and investigate th61e mechanisms leading to its various biological functions. In this study, we performed basic research experiments. For the clinical use of this compound, several issues should be addressed, in terms of synthesis efficiency, cost, and clinical trials; these require our further efforts.

## AUTHOR CONTRIBUTIONS

Jiajun He and Wen Chen conceived and designed the research, performed the experiments and data collection. Xingyu Chen and Yu Xie helped with the data analysis. Yunfeng Lin and Xiaoxiao Cai supervised the project, and reviewed, edited and finalized the manuscript. Jiajun He and Wen Chen contributed equally to this work.

## FUNDING INFORMATION

This study was supported by National Key R&D Program of China (2019YFA0110600), National Natural Science Foundation of China (82171006, 81970986) and Sichuan Province Youth Science and Technology Innovation Team (2022JDTD0021).

## CONFLICT OF INTEREST STATEMENT

The authors declare no conflict of interest.

## Data Availability

The data that support the findings of this study are available from the corresponding author upon reasonable request.
